# The impacts of genetic polymorphisms in genes of base excision repair pathway on the efficacy and acute toxicities of (chemo)radiotherapy in patients with nasopharyngeal carcinoma

**DOI:** 10.18632/oncotarget.20203

**Published:** 2017-08-10

**Authors:** Jing Wang, Chengxian Guo, Xiaochang Gong, Fan Ao, Yuling Huang, Lihua Huang, Yiqiang Tang, Chunling Jiang, Xiaoxue Xie, Qing Dong, Min Huang, Jingao Li

**Affiliations:** ^1^ Department of Radiation Oncology, Jiangxi Cancer Hospital, Nanchang 330029, China; ^2^ Department of Intensive Care Unit, Jiangxi Cancer Hospital, Nanchang 330029, China; ^3^ Center of Clinical Pharmacology, Third Xiangya Hospital, Central South University, Changsha 410013, China; ^4^ Center for Medical Experiments, Third Xiangya Hospital, Central South University, Changsha 410013, China; ^5^ Department of Radiation and Oncology, Hunan Provincial Tumor Hospital and Affiliated Tumor Hospital of Xiangya Medical School, Central South University, Changsha 410013, China; ^6^ Department of Graduate Study, Medical School of Nanchang University, Nanchang 330006, China

**Keywords:** base excision repair (BER) genes, single nucleotide polymorphism (SNP), nasopharyngeal carcinoma (NPC), acute radiation toxicity, short-term efficacy

## Abstract

**Purpose:**

To explore whether polymorphisms in base excision repair (BER) pathway genes are predictors of (chemo)radiotherapy outcome in patients with nasopharyngeal carcinoma (NPC).

**Methods:**

We genotyped five potentially functional single nucleotide polymorphisms (SNPs) of three genes in the BER pathway in 174 NPC patients who were treated with (chemo)radiotherapy. Sequenom MassArray was used for SNPs analysis. The efficacy at the end of radiotherapy and at 3 months after radiotherapy was evaluated by Response Evaluation Criteria in Solid Tumors (RECIST). Acute radiation toxicity was scored using Radiation Therapy Oncology Group and the European Organization for Research and Treatment of Cancer (RTOG/EORTC) acute radiation morbidity scoring criteria. Logistic regression was employed to assess the multivariate analyses.

**Results:**

We found that the wide genotype GG of X-ray repair cross-complementing 1 (*XRCC1*) rs25489 (GG vs GA: OR=3.833, 95%CI=1.512-9.714, *P*=0.005; GG vs GA+AA: OR=3.610, 95%CI=1.496-8.713, *P*=0.004) and the wide genotype CC of 8-oxoguanine DNA glycosylase (*OGG1*) rs1052133 (CC vs GG: OR=0.263, 95%CI=0.073-0.951, *P*=0.042; CC vs CG+GG: OR=0.454, 95%CI=0.195-1.053, *P*=0.066) were positively and negatively associated with primary tumor efficacy at the end of radiotherapy, respectively. By contrast, no association was found between BER gene polymorphisms and the treatment outcomes at 3 months post-treatment or the treatment-related acute toxicities.

**Conclusions:**

The SNPs of the BER genes may act as biomarkers for the curative effect of (chemo)radiotherapy. Further study with long-time follow-up and large population is needed for accurate assessment.

## INTRODUCTION

Nasopharyngeal carcinoma (NPC) is one of the most common malignant tumors in Southern China [1]. Radiotherapy has long been the mainstay of treatment for patients with loco-regionally confined NPC. The 3-year disease-free survival rate of over 80% could be achieved after intensity modulated radiation therapy (IMRT) [2]. However, about 20% of patients still experience either loco-regional or distant treatment failure [2]. The difference of treatment outcomes is partly due to the individual radiosensitivity, especially the ability of DNA damage repair [3–6].

Base excision repair (BER) pathway is the main way to repair the radiation-induced DNA single strand break, including the apurinic/apyrimidinic (AP) site break and DNA base injury [7]. The main enzymes involved in the BER pathway are DNA glycosylase, AP endonuclease, DNA polymerase and DNA ligase [7]. Several key repair genes play important roles in BER pathway, such as the X-ray repair cross-complementing 1 (*XRCC1*), 8-oxoguanine DNA glycosylase (*OGG1*), and apurinic/apyrimidinic endonuclease 1 (*APEX1*) genes, which are associated with human tumor susceptibility and radiation toxicity [8–10]. *XRCC1* can connect and fill the DNA incision in the final stage by forming a complex with poly(ADP-ribose) polymerase, DNA ligase III and DNA polymerase β [11]. *OGG1* is a glycosylase that participates in removing oxidatively induced DNA base lesions [12]. And, *APEX1* can detect and incise the AP sites in the early stage of DNA damage and plays a role in the inflammatory response [13].

Single nucleotide polymorphism (SNP) is a DNA sequence polymorphism caused by single nucleotide change, which could influence the gene expression and protein functions, leading to different susceptibility to disease and affecting the sensitivity of radiotherapy [14–17]. Currently, studies on the association between SNPs of BER pathway genes and radiation reaction were mainly concentrated on breast, prostate and lung cancers [18–20]. *XRCC1* rs3213245 (c.-77T>C) was associated with grade ≥2 acute skin toxicity in breast cancer patients receiving radiotherapy after breast conserving surgery [18]. In prostate cancer patients treated with radiotherapy, *XRCC1* rs25489 (Arg280His) mutation was associated with a lower risk of late bladder and/or rectal toxicity [19]. In patients with non-small cell lung cancer treated with definitive radiation therapy, *XRCC1* rs25487 (A>G) AA genotype was associated with a lower risk of grade ≥ 2 radiation pneumonitis, whereas *APEX1* rs1130409 (T>G) GG genotype was associated with an increased risk of grade ≥ 2 radiation pneumonitis in white population [20]. The SNPs of BER pathway genes have been proved to be associated with radiosensitivity. But there were few researches on the relationship between BER pathway gene SNPs and (chemo)radiotherapy efficacy or acute toxicities in NPC patients. From this point, we intend to clarify the impact of the common functional SNPs in BER pathway genes on (chemo)radiotherapy in patients with NPC.

## RESULTS

### Population characteristics and genotyping

The demographic features of the enrolled patients are summarized in Table 1. 174 patients with nasopharyngeal non-keratinizing carcinoma were used for the final analysis, including 118 men and 56 women, with a mean age of 51 years old (ranging from 14–81 years old) at the time of diagnosis. 140 of the 174 patients (80.5%) were treated with a combination of radiotherapy and platinum based chemotherapy. The overall stage distribution was 13.8% (24/174) for stage I-II and 86.2% (150/174) for stage III-IV by 7^th^ American Joint Committee on Cancer (AJCC) staging system. There were 116 EBV-positive patients and 58 EBV-negative patients before treatments. The results of genotypes of 5 SNPs in *XRCC1*, *OGG1* and *APEX1* genes of the 174 patients are shown in Table 2. All SNPs were analyzed by Hardy-Weinberg equilibrium. However, the genotype distributions of rs25487 and rs3213245 were not in accordance with the Hardy-Weinberg equilibrium and excluded in further analysis.

**Table 1:** General characteristics of patients included in this study

Characteristics	*n* (%)
Gender	
male	118 (67.8)
female	56 (32.2)
Age at diagnosis (years)	
range	14 - 81
mean ± SD	50.55±11.72
BMI	
< 24	115 (66.1)
≥ 24	59 (33.9)
Drinking	
yes	44 (25.3)
no	130 (74.7)
Smoking	
yes	76 (43.7)
no	98 (56.3)
Family history	
yes	24 (13.8)
no	150 (86.2)
T stage^1^	
1-2	45 (25.9)
3-4	129 (74.1)
N stage^1^	
0-1	88 (50.6)
2-3	86 (49.4)
Clinical stage^1^	
I-II	24 (13.8)
III-IV	150 (86.2)
Treatment	
radiotherapy alone	34 (19.5)
chemoradiotherapy	140 (80.5)
EBV-DNA	
negative	58 (33.3)
positive	116 (66.7)

^1^ Using 7^th^ American Joint Committee on Cancer (AJCC) staging system.

BMI, body mass index; SD, standard deviation; EBV-DNA, plasma Epstein-Barr virus DNA.

**Table 2:** Genotype distribution of 5 SNPs in BER pathway genes

Gene	Polymorphic site	Alleles (wild/mutant)	Genotype distribution^1^	HWE
*XRCC1*	rs25487	G/A	65/95/14	**0.010**
*XRCC1*	rs25489	G/A	129/39/6	0.170
*XRCC1*	rs3213245	T/C	109/64/1	**0.009**
*OGG1*	rs1052133	C/G	57/83/34	0.702
*APEX1*	rs1130409	T/G	68/88/18	0.176

^1^Wild homozygote / heterozygote / mutant homozygote.

HWE, Hardy-Weinberg equilibrium.

*P* values < 0.05 are shown in bold.

### Association between gene polymorphisms and short-term efficacy

According to the Response Evaluation Criteria in Solid Tumors 1.1 (RECIST 1.1), at the end of radiotherapy, 140 patients (80.5%) achieved complete remission (CR) of their primary tumors. Of the 144 patients with lymph node metastasis, 125 patients (86.8%) had CR of the metastatic nodes. 128 of the 174 patients had complete clinical and imaging information 3 months after radiotherapy, of which 118 (92.2%) had CR in their primary sites, and 97 (89.8%) of the 108 patients with node metastases before radiotherapy were in CR regionally.

After adjusting for potentially prognostic factors, *XRCC1* rs25489 and *OGG1* rs1052133 had a significant impact on primary tumor efficacy at the end of radiotherapy (Table 3). The likelihood of non-CR was higher in carriers of *XRCC1* rs25489 GA and GA+AA genotypes than that of GG genotype (OR=3.833, 95%CI=1.512-9.714, *P*=0.005; OR=3.610, 95%CI=1.496-8.713, *P*=0.004; respectively). Patients with *OGG1* rs1052133 GG and CG+GG had a lower risk of non-CR than that with CC (OR=0.263, 95%CI=0.073-0.951, *P*=0.042; OR=0.454, 95%CI=0.195-1.053, *P*=0.066).

**Table 3:** Multivariate analyses of the correlation between SNPs and treatment efficacy at the end of radiotherapy

SNPs	Genotypes	Primary tumor efficacy^1^ (*n*=174)					Lymph node efficacy^1^ (*n*=144)				
		CR^2^	Non-CR^2^	OR	95%CI	*P*	CR^2^	Non-CR^2^	OR	95%CI	*P*
*XRCC1* rs25489	GG:GA	111/25/4	18/14/2	3.833	1.512-9.714	**0.005**	95/26/4	15/4/0	0.818	0.199-3.358	0.780
	GG:AA			2.552	0.360-18.088	0.348			-	-	-
	GG:(GA+AA)			3.610	1.496-8.713	**0.004**			0.699	0.174-2.813	0.615
	(GG+GA):AA			1.764	0.257-12.110	0.564			-	-	-
*OGG1* rs1052133	CC:CG	42/68/30	15/15/4	0.552	0.227-1.344	0.191	45/58/22	5/9/5	1.922	0.518-7.128	0.329
	CC:GG			0.263	0.073-0.951	**0.042**			2.832	0.605-13.252	0.186
	CC:(CG+GG)			0.454	0.195-1.053	0.066			2.161	0.629-7.430	0.221
	(CC+CG):GG			0.371	0.114-1.209	0.100			1.895	0.532-6.745	0.324
*APE(X)1* rs1130409	TT:TG	51/74/15	17/14/3	0.853	0.348-2.088	0.728	47/65/13	9/8/2	0.592	0.179-1.963	0.392
	TT:GG			0.392	0.088-1.749	0.220			0.698	0.107-4.570	0.708
	TT:(TG+GG)			0.719	0.310-1.667	0.442			0.615	0.203-1.865	0.390
	(TT+TG):GG			0.420	0.099-1.786	0.240			0.862	0.139-5.331	0.873

^1^ Adjusted for age, gender, drinking, smoking, family history, BMI, TNM stage, clinical stage, treatment and EBV-DNA.

^2^ Wild homozygote / heterozygote / mutant homozygote.

SNPs, single nucleotide polymorphisms; CR, complete remission; OR, odds ratio; 95%CI, 95% confidence interval.

*P* values < 0.05 are shown in bold.

No relationship between SNPs and the treatment efficacy at 3 months after radiotherapy was found in our research (Table 4).

**Table 4:** Multivariate analyses of the correlation between SNPs and treatment efficacy at 3 months after radiotherapy

SNPs	Genotypes	Primary tumor efficacy^1^ (*n*=128)					Lymph node efficacy^1^ (*n*=108)				
		CR^2^	Non-CR^2^	OR	95%CI	*P*	CR^2^	Non-CR^2^	OR	95%CI	*P*
*XRCC1* rs25489	GG:GA	84/28/6	7/3/0	1.351	0.243-7.502	0.731	71/22/4	8/3/0	1.150	0.225-5.881	0.866
	GG:AA			-	-	-			-	-	-
	GG:(GA+AA)			1.032	0.196-5.425	0.970			1.052	0.208-5.310	0.951
	(GG+GA):AA			-	-	-			-	-	-
*OGG1* rs1052133	CC:CG	38/58/22	6/4/0	0.535	0.122-2.334	0.405	35/44/18	2/5/4	1.838	0.296-11.401	0.514
	CC:GG			-	-	-			3.795	0.555-25.975	0.174
	CC:(CG+GG)			0.335	0.079-1.427	0.139			2.446	0.455-13.154	0.297
	(CC+CG):GG			-	-	-			2.617	0.588-11.656	0.207
*APE(X)1* rs1130409	TT:TG	40/65/13	5/4/1	0.480	0.096-2.407	0.372	32/55/10	5/4/2	0.574	0.118-2.802	0.493
	TT:GG			0.479	0.037-6.166	0.572			1.116	0.141-8.859	0.917
	TT:(TG+GG)			0.479	0.106-2.166	0.339			0.702	0.171-2.875	0.623
	(TT+TG):GG			0.666	0.057-7.756	0.745			1.375	0.184-10.300	0.756

^1^ Adjusted for age, gender, drinking, smoking, family history, BMI, TNM stage and clinical stage, treatment and EBV-DNA.

^2^ Wild homozygote / heterozygote / mutant homozygote.

SNPs, single nucleotide polymorphisms; CR, complete remission; OR, odds ratio; 95%CI, 95% confidence interval.

*P* values < 0.05 are shown in bold.

### Association between gene polymorphisms and acute radiation toxicity

According to the Radiation Therapy Oncology Group and the European Organization for Research and Treatment of Cancer (RTOG/EORTC) acute radiation morbidity scoring criteria, there were 30, 61, 65 and 18 patients who had exhibited radiation-induced mucositis of grade 1, 2, 3 and 4, respectively. As shown in Table 5, no SNP in BER pathway genes were associated with the risk of acute mucositis when severe mucositis (G3+, *n*=83) were compared with moderate or less mucositis (G0-2, *n*=91).

**Table 5:** Multivariate analyses of the correlation between SNPs and radiation toxicity in NPC patients (*n*=174)

SNPs	Genotypes	Radiation mucositis^1^					Radiation dermatitis^1^				
		G0-2 ^2^	G3+ ^2^	OR	95%CI	*P*	G0-1 ^2^	G2+ ^2^	OR	95%CI	*P*
*XRCC1* rs25489	GG:GA	68/19/4	61/20/2	1.222	0.572-2.615	0.605	96/27/5	33/12/1	1.373	0.592-3.184	0.460
	GG:AA			0.517	0.082-3.246	0.481			0.926	0.090-9.514	0.948
	GG:(GA+AA)			1.093	0.533-2.244	0.808			1.322	0.587-2.979	0.500
	(GG+GA):AA			0.494	0.079-3.084	0.451			0.850	0.084-8.625	0.891
*OGG1* rs1052133	CC:CG	30/40/21	27/43/13	1.199	0.595-2.414	0.611	42/58/28	15/25/6	1.119	0.503-2.492	0.782
	CC:GG			0.719	0.295-1.753	0.467			0.521	0.172-1.576	0.248
	CC:(CG+GG)			1.033	0.536-1.993	0.922			0.910	0.426-1.944	0.808
	(CC+CG):GG			0.646	0.293-1.423	0.278			0.487	0.179-1.323	0.158
*APE(X)1* rs1130409	TT:TG	39/45/7	29/43/11	1.299	0.652-2.586	0.457	51/64/13	17/24/5	1.341	0.605-2.976	0.470
	TT:GG			1.787	0.577-5.538	0.314			0.875	0.234-3.273	0.843
	TT:(TG+GG)			1.379	0.716-2.656	0.336			1.235	0.580-2.630	0.584
	(TT+TG):GG			1.569	0.535-4.603	0.412			0.761	0.217-2.673	0.670

^1^ Adjusted for age, gender, drinking, smoking, family history, BMI, TNM stage and clinical stage, treatment and EBV-DNA.

^2^ Wild homozygote / heterozygote / mutant homozygote.

SNPs, single nucleotide polymorphisms; OR, odds ratio; 95%CI, 95% confidence interval.

*P* values < 0.05 are shown in bold.

Acute radiation dermatitis developed in all of the 174 patients, including grade 1 in 128 patients (73.6%), grade 2 in 31 patients (17.8%), and grade 3 in 15 patients (8.6%). There was no grade 0 or grade 4 dermatitis observed during the treatment. There were no significant correlation between SNPs in BER pathway genes and the severity of radiation dermatitis when G0-1 dermatitis was compared with G2+ dermatitis after a multivariate adjustment (Table 5).

### Relation between radiation dose to specific region and radiation toxicity

The relation between radiation toxicity and the mean radiation doses to specific organs/tissues, including the oral cavity, larynx, skin and 4mm under the skin (skin_4mm_), was investigated in this study. As shown in Table 6, There were no significant differences of radiation dose to the oral cavity (3893.65 ± 545.20 vs. 3930.05 ± 551.43, *P*=0.662) and larynx (4215.65 ± 350.68 vs. 4215.06 ± 325.32, *P*=0.991) between the patients with G0-2 and G3+ mucositis. On the other hand, radiation doses to the skin (3496.06 ± 722.31 vs. 3245.97 ± 583.61, *P*=0.086) and skin_4mm_ (3656.47 ± 462.32 vs. 3577.97± 475.70, *P*=0.509) were also not significantly different between the patients with G0-1 and G2+ dermatitis.

**Table 6:** Comparison of radiation doses between patients with low and high radiation toxicity

	Radiation mucositis				Radiation dermatitis			
	Oral cavity		Larynx		Skin^1^		Skin_4mm_^2^	
	G0-2	G3+	G0-2	G3+	G0-1	G2+	G0-1	G2+
*n*	91	83	91	83	86	31	68	20
Radiation dose (mean±SD, cGy)	3893.65±545.20	3930.05±551.43	4215.65±350.68	4215.06±325.32	3496.06±722.31	3245.97±583.61	3656.47±462.32	3577.97±475.70
*P*	0.662		0.991		0.086		0.509	

^1^ The mean dose of skin was calculated in only 117 patients when the radiotherapy plan was performed.

^2^ The mean dose of skin_4mm_ was calculated in only 88 patients when the radiotherapy plan was performed.

SD, standard deviation.

*P* values < 0.05 are shown in bold.

## DISCUSSION

In our study, we found that the wide genotype GG of *XRCC1* rs25489 and the wide genotype CC of *OGG1* rs1052133 were positively and negatively, respectively, associated with primary tumor efficacy at the end of radiotherapy. By contrast, no association was found between the BER gene polymorphisms and both tumor responses at 3 months after treatment and treatment related toxicities.

BER pathway gene polymorphisms were reported to be related to the oncogenesis [21–23]. Recent studies also showed that the mutation of BER pathway genes could increase the radiosensivity by reducing the DNA repair ability [24]. Costa et al analyzed the association of common polymorphisms in BER pathway genes with the risk and prognosis of oropharyngeal squamous cell carcinoma (OPSCC) [10]. By comparing 200 OPSCC patients with 200 controls, they found that *XRCC1* rs1799782 CT+TT genotypes (19.5% vs 11.0%, *P*=0.04) and *XRCC1* T-T-G-G (for rs3213245, rs1799782, rs25489 and rs25487) haplotype (17.5% vs 10.0%, *P*=0.04) were more common in patients with OPSCC than in controls. They also analyzed 125 patients with stage IV disease and found that patients with *OGG1* rs1052133 CC genotype had significant shorter progression free survival (PFS) than those with CG+GG genotypes (HR=1.68, *P*=0.02). Our data showed that patients with the wild genotype CC of *OGG1* rs1052133 had a high risk of non-CR of the primary tumors at the end of radiotherapy, which was consistent with Costa et al’s research. But there was no association between this SNP and the efficacy at 3 months after radiotherapy. Since there are lots of factors that participate in the damage mechanism of radiation, such as DNA repair after damage, cell proliferation, apoptosis and necrosis, inflammation, and oxidative stress [17]. With the progress of the disease, the influence of the above factors in each phase may be different. Moreover, there had been some patients (46 patients) who withdrew in the process of follow-up, which might be one of the reasons for the inconsistent results. Our study only explored the short-term efficacy, and the long-term curative effect still needs further follow-up.

Jin et al found that heavy smokers (>20 pack-years) carrying *XRCC1* rs25487 GG genotype had significantly lower PFS rates than others in the 75 patients with NPC at stage II-IVA-B (HR=2.019, 95%CI: 1.010-4.036, *P*=0.047) [25]. In 60 Chinese patients with locally advanced NPC after radiotherapy, Zhai et al discovered that *XRCC1* rs25487 AA was correlated with higher rate of medium-term tumor regression after RT for primary nasopharyngeal neoplasm and metastatic lymph nodes than other two genotypes (>80% vs 40-60%, *P* < 0.01) [26]. In our study, *XRCC1* rs25487 was not in accordance with the Hardy-Weinberg equilibrium and thus excluded in further analysis. However, it still needs a large number of samples to validate.

Several studies showed that BER pathway related gene polymorphisms were associated with acute or chronic radiation toxicities in cancer patients. In a study of 101 patients with head and neck squamous cell carcinoma, the development of grade ≥2 mucositis was increased in patients with *XRCC1* rs25487 A allele (HR=1.72) [27]. Alsbeih et al analyzed the association between *XRCC1* rs25487 (g.G28152A) and the late reaction to radiotherapy in 60 NPC patients, and found that *XRCC1* g.28152A allele was significantly associated with a lower grade condition of grade ≥2 skin and deep tissue fibrosis (OR=0.30, 95%CI: 0.10-0.89, *P*=0.02) [28]. After increasing the sample size to 155 patients, they still found that compared with wild genotypes, patients with *XRCC1* rs25487 A allele had a lower risk of grade 3-4 fibrosis [29]. Another research of 114 NPC patients proved that the *XRCC1* rs25487 GA genotype was significantly associated with the development of grade 3 dermatitis (OR=2.65, 95%CI=1.04-6.73, *P*=0.037) and was also associated with higher incidence of grade 3 mucositis with a borderline statistical significance (OR=2.11, 95%CI=0.951-4.66, *P*=0.065) [30]. However, Zhai et al did not find any association between *XRCC1* rs25487 and acute or chronic radiation toxicities in 60 patients with locally advanced NPC [26]. We did not find any BER gene polymorphism that has significant impact on acute radiation toxicity reaction in NPC patients. This may be because that the radiation damage to normal tissues, such as skin and mucosa damage, depends mainly on cell regeneration, proliferation and inflammation, while the role of DNA damage repair is relatively small.

We didn’t find any relations between the mean dose to the oral cavity and skin and radiation mucositis or dermatitis. One reason is that the prescribed doses to the normal tissues were strictly limited, and the radiation doses to the normal tissues were only slightly different among this cohort of patients. An other reason for this paradoxical result is the inaccuracy of delineation of the normal tissues. There was often compromise in the contouring of normal tissues if the tumor was close to or even invaded the normal tissues. In this case, only the tissues not involved by tumors were contoured and protected in an effort to best treat the tumors, and the mean dose to the normal tissues could be under-estimated.

In summary, our study explored the impact of polymorphisms in BER pathway genes on (chemo)radiotherapy in NPC patients. We only found the BER SNPs had significant correlation with the curative effect at the end of radiation therapy and did not find any influence on radiation toxicity. Further study with long-time follow-up and large population would be needed to explain the relevance between polymorphisms and the long-term survival.

## MATERIALS AND METHODS

### Patient selection and treatment

A total of 174 NPC patients were recruited into our research between April 2014 and September 2015 in Jiangxi Cancer Hospital, China, according to the inclusion and exclusion criteria. Patients were included in the study if they had pathologically confirmed non-keratinizing NPC if they met all of the following criteria: no distant metastasis at diagnosis; previously untreated of their tumors; ECOG performance status score ≤ 1; no severe comorbilities of heart, lung, liver or kidney. Lactating femal patients or patients with pregnancy, patients with active infections before treatment and history of any other tumors were excluded from the study. The study protocol was approved by the Ethics Committee of Jiangxi Cancer Hospital and registered online through the website of Chinese Clinical Trial Registry (ChiCTR-OPC-14005257). All eligible patients signed the informed consent before treatment.

All the patients were treated by radiotherapy with 6 MV photons by use of intensity-modulated radiation therapy (IMRT). The radiation techniques were reported elsewhere [31]. Briefly, the prescription doses were 70 Gy in 30 to 33 fractions to the primary tumors, 66 to 70 Gy in 30 to 33 fractions to the positive cervical lymph nodes, and 54 to 60 Gy in 30 to 33 fractions to other areas with potential tumor involvement. All radiotherapies were delivered once daily, 5 days a week. 34 of the 174 patients were treated by radiotherapy alone, while other patients received a combination of radiotherapy and platinum-based chemotherapy.

Demographic and clinical data, such as age, gender, smoking and drinking status, TNM stage (using 7^th^ AJCC staging system), MRI and nasopharyngoscope information, were recorded. Those who had smoked >100 cigarettes in their lifetime were considered “smoker”, and all others were considered “non-smoker” [32]. “Drinker” was defined as a daily ethanol intake > 40 g for men and > 20 g for women [33]. Additionally, the mean doses to the oral cavity, larynx, skin and 4mm under the skin (skin_4mm_) in the radiotherapy plans were also collected.

### Study end points

Acute radiation toxicity was evaluated from the first day to the end of radiotherapy. In our study, radiation-induced mucositis and dermatitis were evaluated and scored according to RTOG/EORTC acute radiation morbidity score criteria [34, 35]. Patients with high grade of toxicity were compared with patients with low toxicity.

The efficacy at the end of radiotherapy and at 3 months after radiotherapy was evaluated by RECIST 1.1 [34, 36] based on MRI imaging and nasopharyngoscope. The RECIST 1.1 defines the efficacy with complete remission (CR), partial remission (PR), stable disease (SD) and progression disease (PD). The primary tumor efficacy is only for the tumor. In this study, we divided all patients into two groups: CR or non-CR.

### SNP selection and genotyping analysis

The National Center for Biotechnology Information dbSNP database (http://www.ncbi.nlm.nih.gov/) and HapMap database (http://hapmap.ncbi.nlm.nih.gov/) were used to identify the potentially functional SNPs based on the following 3 criteria: 1) location in the promoter untranslated region or coding region of gene; 2) a minor allele frequency (MAF) ≥ 10% in Han Chinese reported in the dbSNP database; 3) previous reports of an association with radiosensitivity in NPC or other cancers. According to the candidate gene approach, a total of 5 SNPs in 3 BER pathway genes were genotyped, including *XRCC1* (rs25487, rs25489 and rs3213245), *OGG1* (rs1052133) and *APEX1* (rs1130409).

Peripheral venous blood (3 ml) was collected from patients before radiotherapy and stored at −20°C. Genomic DNA was extracted from the blood lymphocytes using a Wizard^®^ Genomic DNA Purification Kit (Promega, America) according to the manufacturer’s instructions. All the DNA samples were stored at −80°C until genotyping analysis. Sequenom MassArray (BioMiao Biological, Beijing, China) was used for SNPs analysis.

### Statistical analysis

Statistical analysis was performed using SPSS 18.0 software (IBM, Armonk, New York, USA) and *P*<0.05 was considered significant. The patients’ characteristics, including gender, age, body mass index (BMI), smoking history, drinking history, family history, TNM stage, use of chemotherapy and EBV, were chosen as adjusting factors for multivariate analysis. The multivariate analysis of the association between SNPs and radiation toxicity or short-term efficacy was estimated by computing the odds ratio (OR) and 95% confidence interval (95%CI) from logistic regression analyses.
